# Effect of Perceived Social Support on Mortality Rate Among Older Chinese Americans

**DOI:** 10.1093/geroni/igaa057.642

**Published:** 2020-12-16

**Authors:** Charu Verma, Mengting Li, XinQi Dong

**Affiliations:** 1 Rutgers University--New Brunswick, New Brunswick, New Jersey, United States; 2 Rutgers University, New Brunswick, New Jersey, United States

## Abstract

Most existing studies have examined the relationship between social support and health in cross-sectional data. However, the changing dynamics of social support over time and its relationship with all-cause mortality have not been well explored. Using data from the Pine Study (N = 3,157), this study examined whether social support was associated with time of death at an 8 years follow-up among older Chinese Americans. Social support from a spouse, family members and friend were collected at the baseline using an HRS social support scale. Perceived social support and time of death were ascertained from the baseline through wave 4. Cox proportional hazard models were used to assess associations of perceived support with the risk of all-cause mortality using time-varying covariate analyses. Covariates included age, sex, education, income, and medical comorbidities. All study participants were followed up for 8 years, during which 492 deaths occurred. In multivariable analyses, the results showed that positive family support [HR 0.91; 95% CI (0.86, 0.98)] and overall social support [HR 0.95; 95% CI (0.92,0.98)] were significantly associated with a lower risk of 8-year mortality. Results demonstrate robust association in which perceived positive family and overall social support over time had a protective effect on all-cause mortality risk in older Chinese Americans. Interventions could focus on older adults with low social support and protect their health and well-being. Future studies could further explore why social support from family is different from social support from other sources regarding mortality risk in older Chinese Americans.

